# Latent, Lytic, and Linked to Multiple Sclerosis—How EBV Drives Autoimmunity

**DOI:** 10.1002/eji.70153

**Published:** 2026-02-22

**Authors:** Fabienne Läderach, Elena Bremer, Christian Münz

**Affiliations:** ^1^ Institute of Experimental Immunology University of Zürich Zürich Switzerland; ^2^ Viral Immunobiology, Institute of Experimental Immunology University of Zürich Switzerland

**Keywords:** Autoimmunity, B cells, CNS, Epstein‐Barr virus, Multiple‐Sclerosis

## Abstract

Epstein–Barr virus (EBV) is a human tumor virus best known for its B cell‐transforming capacity and association with lymphomas and carcinomas. Epidemiological studies have suggested that its infection, in addition, is necessary for the development of the autoimmune disease multiple sclerosis (MS). The very same oncogenes that drive EBV‐associated malignancies might also induce differentiation of B cell subsets that initiate neuroinflammation. This review will discuss how insufficient immune control might allow for sizeable populations of T‐bet^+^CXCR3^+^ B cells to infiltrate the central nervous system (CNS), attract other lymphocytes, efficiently stimulate T cells in the CNS, and differentiate into antibody‐producing plasma cells, thereby contributing to inflammation and autoantibody production in a subset of MS patients. This CNS‐infiltrating B‐cell population could be targeted by EBV‐specific treatments to complement existing MS therapies.

## Graphical abstract

EBV latent and lytic genes uniquely contribute to Multiple Sclerosis development by various immunopathogenic mechanisms in susceptible hosts. Briefly, EBV infection shapes the B‐cell repertoire and promotes the oligoclonal expansion and subsequent CNS infiltration of T‐bet^+^CXCR3^+^ B cells, resulting in a cascade of immune dysregulation, CNS inflammation, and autoreactive T cell stimulation. Created in BioRender. Läderach, F. (2026) https://BioRender.com/b15duil.

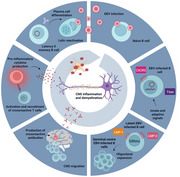

## 1 Introduction to the Dual Life Cycle of the Epstein–Barr Virus

Discovered in 1964 within Burkitt's lymphoma (BL) cells, Epstein–Barr virus (EBV) is one of the eight known human herpesviruses. With its approximately 172kb genome, EBV is a relatively large double‐stranded DNA γ_1_‐herpesvirus [1]. EBV is primarily transmitted through saliva exchange, with the majority of individuals contracting the virus asymptomatically within the first years of life. However, when primary virus infection is delayed until adolescence or adulthood, as is often the case in more developed countries, it can manifest as infectious mononucleosis (IM) [2], an acute but typically self‐resolving lymphoproliferative disorder [3]. During primary infection, EBV initially targets cells within the submucosal secondary lymphoid tissues, where it gains access to its main cellular reservoir, the host B cells. Once within the host's B cells, EBV, similar to other herpesviruses, has a lytic and latent life cycle.

During the lytic cycle, virions are produced, ultimately leading to the lysis of the infected cell. In this phase, viral gene expression follows a temporal cascade categorized into immediate‐early, early, and late phases. Immediate‐early genes, such as BZLF1 and BRLF1, function as transcriptional activators that initiate expression of early genes involved in viral DNA replication [4]; following this, late genes support the formation of viral particles [5]. Analysis of EBV gene expression during primary B cell infection demonstrated that immediate‐early and early lytic genes are expressed within the first few days postinfection, while some viral proteins are directly delivered by the incoming virions [5].

The latent cycle allows EBV to persist long‐term within the host without producing viral particles. Despite the absence of virion production, continuous proliferation of infected B cells during early latency allows for viral genome maintenance and replication. Following primary infection, latency eventually becomes the default program after viral DNA circularization to episomes in the nucleus and appears to be sufficient for viral spread within the host. In contrast, the lytic cycle is likely more crucial for transmission between hosts [6]. Latency is classified into different stages characterized by a restrictive gene expression profile that primarily promotes B cell activation, proliferation, and resistance to cell death. Latency III is characterized by the expression of three latent membrane proteins (LMP1, LMP2A, LMP2B), six nuclear antigens (EBNA1, EBNA2, EBNA3A, EBNA3B, EBNA3C, and EBNA‐LP), as well as noncoding RNAs including micro‐RNAs (miRNAs) and EBV‐encoded small RNAs (EBERs) [7], which promote B cell survival and proliferation, and facilitates their germinal center (GC) B cell‐like differentiation [8]. Following entry into this differentiation, EBV‐infected B cells further downregulate viral genes and enter latency II, characterized by the expression of only four latent proteins: EBNA1, LMP1, LMP2A, and LMP2B. This expression pattern ensures survival during this differentiation and promotes maturation toward the memory B cell phenotype [6]. During latency I, memory B cells transiently express EBNA1, which is crucial for the amplification and retention of viral episomes during cell division [9]. Once the memory B cells enter a quiescent state, referred to as latency 0, only EBERs and some miRNAs are detected. In latency 0, EBV can persist within the host for life [10], and stimulation of the B cell receptor during latency 0 or I can trigger lytic reactivation [11]. Plasma cell differentiation induces the expression of the early lytic genesBZLF1 and BRLF1, which induce a transcriptional cascade leading to the expression of lytic genes essential for virion production and transmission, thereby starting the cycle of primary infection anew [12].

## 2 Adaptive Immune Control of Persistent EBV Infection

To prevent viral reactivation and uncontrolled proliferation of infected cells, it is essential that the infected host mounts robust and continuous immune surveillance [13]. EBV‐specific CD8^+^ T cells, which make up a large proportion of the human antiviral memory T cell pool and can persist stably for decades, constitute the key players in the ongoing immunological dance between EBV and host defences [14]. These cells are mainly directed against immediate‐early and early EBV lytic cycle proteins such as BZLF1 and BMRF1 [15], as well as latent antigens in the EBNA3 family (EBNA3A, EBNA3B, EBNA3C), and, at a lower frequency, LMP2 [14]. In the acute phase of symptomatic EBV infection, the CD8^+^ T cell repertoire directed against early lytic epitopes can constitute over 30% of total CD8^+^ T cells [16]. Responses directed against latent epitopes are initially rarer but increase in frequency after the resolution of the initial infection [13, 16]. CD8^+^ T cells directed against EBNA3‐derived peptides effectively eliminate latently infected B cells through perforin and granzyme B secretion and thus limit lytic reactivation [13].

CD4^+^ T cells complement this response by supporting CD8^+^ T cell activation, proliferation, and memory formation, as well as providing T cell help to B cells and thereby promoting the production of high‐affinity neutralizing antibodies [13]. CD4^+^ T cells have also been shown to directly kill EBV‐infected B cells in response to antigens presented on major histocompatibility complex (MHC) class II molecules; these antigens include peptides derived from both lytic proteins such as BHRF1 [17], BALF4 [18] and BLLF1 [18], and latent proteins such as EBNA2, EBNA3C, and LMP2 [19] as well as from the EBV capsid protein BORF1 [20]. Furthermore, CD4^+^ T cells play a crucial role in recognizing the latent EBV protein EBNA1 [21], which limits its own proteasomal processing and protein translation, and thus renders latency I‐infected B cells nearly invisible to CD8^+^ T cells [22]. Only individuals expressing certain human leukocyte antigen (HLA) class I alleles, such as HLA‐B35:01 or HLA‐A02:03, have CD8^+^ T cells capable of recognizing EBNA1‐derived peptides [23].

Beyond T cells, B cells have dual functions in the context of EBV infection. While memory B cells are the primary site of EBV persistence and serve as a long‐term reservoir for EBV throughout the life of the host [24], they also actively participate in the antiviral immune response by generating virus‐specific antibodies that can neutralize the infection of B cells and epithelial cells [13, 25], thus limiting initial infection and viral spread within the host.

## 3 Epidemiological Evidence for an Association of Delayed Primary EBV Infection With Multiple Sclerosis

EBV's latent life cycle enables it to be a successful tumor virus, with it being associated with around 1.3‐1.9% of tumor cases worldwide, including not only B, but also T and NK cell cancers and various epithelial carcinomas [25]. More recently, the EBV—multiple sclerosis (MS) autoimmunity axis is emerging as a prominent but lesser‐known side effect of dysfunctional EBV control.

MS is a chronic neuroinflammatory disease and is among the most common causes of nontraumatic disability in young adults. MS is characterized by lesions comprised of inflammatory leukocytes in the CNS, including T and B cells, resulting in the immune‐mediated demyelination of the neurons, ultimately leading to progressive neurological deficits [26]. Both genetic and environmental factors are thought to play a role in MS etiology and progression; however, the exact interplay remains incompletely understood. Of the environmental risk factors, infectious pathogens, specifically EBV, have been suggested to play a significant role in MS development and disease progression [26].

One line of evidence for this is provided by migration studies. Migration studies highlight a critical age‐window in which exposure to environmental factors modifies MS risk; children under the age of 15 who move from a high‐ to a low‐prevalence MS region inherit the lower risk of their new environment, while migrating individuals above 15 years of age retain the MS risk of their birthplace [27, 28]. This age‐window may reflect a time‐sensitive period of exposure to environmental risk factors for MS, such as the timing of primary EBV infection. Sero‐epidemiological data show that in low‐income countries, which have lower MS prevalence rates [29], EBV is typically acquired asymptomatically in early childhood [30] and is associated with a lower lifelong MS risk [31]. In industrialized countries, primary EBV infection is frequently delayed to mid‐adolescence and commonly manifests as IM^2^, which is accompanied by a markedly increased risk of developing MS [31]. However, it is impossible to disentangle the effect of the timing of primary EBV infection from other environmental risk factors, such as UV radiation, when considering migration studies alone, since many low‐income countries are situated closer to the equator and tend to receive higher sun exposure compared with higher‐income countries [32]. As such, some researchers argue that the significant latitudinal distribution of MS [33] may be primarily driven by sun exposure [33], while others suggest that this distribution is associated with variation in EBV exposure [34, 35].

Nevertheless, these findings contribute to a growing body of evidence implicating pathogens in MS disease development. Initial findings dating back to 1983 substantiate a link between EBV and MS; increased serum levels of EBV‐specific viral capsid antigen IgG and a higher prevalence of EBV infection were reported in MS patients compared with healthy individuals [35]. More recent studies investigating various environmental risk factors for MS have further solidified the link between EBV infection and the disease's development [36]. Specifically, anti‐EBNA seropositivity [37] and a history of IM [38] stand out as significant risk factors, whereas EBV‐seronegative individuals have a 10‐fold lower risk to develop MS compared with peers infected with EBV in early childhood, and a 20‐fold lower risk compared with individuals with a history of IM [39]. The strongest epidemiological evidence comes from a 2022 longitudinal study that followed 10 million active US military personnel over 20 years, during which time 955 individuals developed MS. The study reported a 32‐fold increased risk of MS following EBV infection [40].

The exact mechanisms by which EBV contributes to MS pathogenesis are not yet fully understood; however, the main hypotheses are believed to be defective immune control of EBV reactivation leading to MS relapses [41], accumulation of central nervous system (CNS) autoantigen cross‐reactive EBNA1‐specific T helper (Th) 1 cells [42], bystander damage [43], as well as molecular mimicry between EBV and CNS autoantigens [44], which could initiate or sustain autoimmunity. Notably, patients with MS show distinct patterns of dysregulated immune responses to EBV [45]. For example, increased levels of EBNA1‐specific IgG antibodies are consistently found in MS patients compared with healthy controls [46], indicating a dysregulated T and B cell response to persistent antigen exposure. Recently, the link between EBV and the clonal expansion of T‐bet^+^ B cells [47] has been brought forward as an important mechanism that links the contribution of EBV to MS pathogenesis. Given EBV's ubiquity in the world's adult population (≥90% infected) [2], its role in MS etiology is likely that of a necessary but not sufficient environmental risk factor; more specifically, EBV is thought to exploit genetic susceptibility [48], creating a permissive environment for MS development.

## 4 T‐bet^+^ B Cells as a Link Between EBV and MS

T‑bet^+^ B cells represent a functionally distinct subset of memory B cells that are present at low levels in healthy individuals but accumulate with age and are expanded in the context of chronic viral infections, including gammaherpesvirus‐68 (γHV68) [49], a murine γ_2_‐herpesvirus. Historically, EBV was thought to infect only naïve B cells in the subepithelial region of the tonsils, where the initiation of GCs results in EBV‐infected memory B cells. Intriguingly, recent evidence demonstrates that EBV can infect memory T‐bet^+^ B cells de novo in vitro [50], and γHV68 infection in mice leads to a significant expansion of this population [49]. This specific phenotype of memory B cell is also found to be enriched in various autoimmune diseases, such as MS [51], systemic lupus erythematosus (SLE) [52], and rheumatoid arthritis (RA) [53], for which a contribution of EBV has been discussed. The differentiation toward this phenotype of T‐bet^+^ memory B cell is induced by the convergence of various innate and adaptive immune signals, namely B cell receptor (BCR) engagement, toll‐like receptor (TLR) 7/9 activation, and exposure to Th1 and follicular helper T cell cytokines such as interferon‐γ (IFN‐γ) and interleukin‐21 (IL‐21) [54]. The expression of T‐bet in B cells results in an increased propensity for IgG1/IgG3 isotype class switching [55] and expression of the chemokine receptor CXCR3 [56], which directs the cells toward inflammatory chemokine gradients (CXCL9/10/11), facilitates tissue infiltration, and thus contributes to the accumulation of T‐bet^+^ B cells at sites of active disease and inflammation [57]. Functionally, T‐bet^+^ B cells are characterized by a distinct pro‐inflammatory cytokine profile and potent antigen‐presenting capacity, due to enhanced expression of the co‐stimulatory molecules CD80 and CD86 in combination with higher levels of MHC class II [58, 59].

**FIGURE 1:**
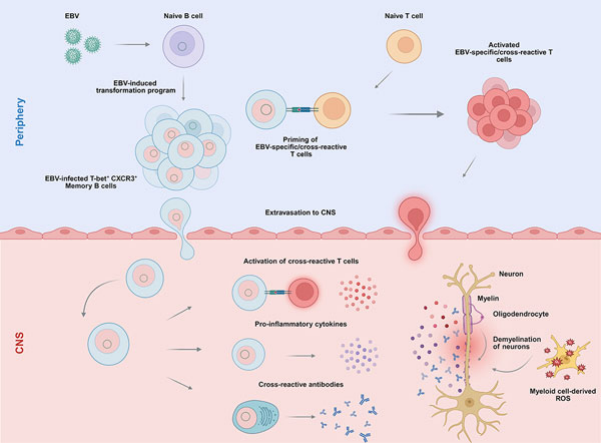
EBV‐driven immunopathogenic mechanisms promoting CNS inflammation. EBV infection drives B cell expansion, differentiating in part into memory T‐bet+ B cells (denoted by a red nucleus) that can home to the CNS. T cells that are primed during EBV infection can be attracted to the CNS by these EBV‐infected T‐bet+ B cells. T‐bet+ B cell interaction with T cells might produce pathogenic inflammation during MS and promote the differentiation of T‐bet+ B cells into plasma cells, leading to auto‐ or poly‐ reactive antibody production that could cause demyelination and neuroaxonal damage. CNS‐resident microglia or CNS‐infiltrating monocyte‐derived cells may be activated by this ongoing immune response, producing reactive oxygen species (ROS) that further amplify inflammation and drive neurodegeneration. Created in BioRender. Läderach, F. (2026) https://BioRender.com/o9v3row.

T‐bet^+^ B cells are thought to contribute to autoimmunity through various mechanisms; these include rapid differentiation to plasma cells for autoantibody production, enhanced antigen presentation, and the secretion of pro‐inflammatory cytokines, such as IFN‐γ, tumor necrosis factor (TNF), and IL‐17 [54]. These effects are not mutually exclusive, and depending on the disease context, some effects may be more pronounced than others. In SLE, T‐bet^+^ B cells are a major source of autoantibodies, with murine models further providing evidence that T‐bet^+^ B cells are also critical for the formation of GCs and that deletion of T‐bet in B cells resulted in reduced mortality and tissue damage [59]. In MS, T‐bet^+^ memory B cells are enriched in the CNS [60] where they are thought to contribute to inflammation [61]and immune cell recruitment [62]. In the CNS, these cells may function as potent antigen‐presenting cells (APCs), driving autoreactive T cell activation, resulting in the maintenance of inflammation in the CNS [60, 61] (Figure 1).

In theory, EBV is well equipped to promote T‐bet^+^ memory B cell differentiation by the expression of the latency proteins LMP1 and LMP2A, which mimic CD40 [63] and BCR^6^ signaling, respectively. During chronic active EBV infection, viral nucleic acids engage TLR9 and stimulate IFN‐α/βsecretion [64]; these pathways represent the prototypical combination of signals known to be crucial for T‐bet^+^ B cell differentiation, thus implicating EBV as a potential driver of this program [65, 66]. Impaired clearance of EBV lytic reactivation may lead to continuous IFN production by lytic viral antigen‐specific T cell populations, as well as persistent BCR and TLR9 signaling, which reflects an environment where T‐bet^+^ B cells could accumulate [67].

### 4.1 Immune Control of EBV Drives Lymphocyte Infiltration Into the Central Nervous System

Despite the absence of elevated blood viral loads [68], MS patients display increased levels of EBNA1 antibodies and higher frequencies of EBNA1‐specific T cells, with the potential to cross‐react with CNS autoantigens [44]. Notably, EBV‐specific CD4^+^ T cell clones in MS patients have been shown to cross‐react with myelin antigens, with EBNA1‐specific clones more frequently recognizing peptides derived from Myelin basic protein (MBP), Myelin proteolipid protein (PLP), and Myelin oligodendrocyte glycoprotein (MOG) than the control autoantigen proinsulin [44]. Although cross‐reactivity in EBV‐specific CD8^+^ T cells has not yet been demonstrated, their T cell receptors were found to be enriched in MS patients, indicating an ongoing anti‐EBV immune response [69]. Molecular mimicry between EBV antigens and CNS autoantigens may arise as a consequence of deficient immune control, where increased viral antigen load drives lower‐affinity, polyreactive, and cross‐reactive immune responses that contribute to CNS autoimmunity in MS.

Poor control of EBV infection could be particularly pronounced in individuals carrying the main genetic risk factor for MS, the MHC class II molecule HLA‐DRB1*15:01 (HLA‐DR15). Individuals with the HLA‐DR15 haplotype display elevated EBNA1 antibody levels and show an increased risk to develop MS even at lower EBNA1 reactivity [70]. Supporting this, our lab recently demonstrated that in a humanized mouse model of EBV infection, mice reconstituted with HLA‐DR15‐positive hematopoietic stem cells (HSCs) exhibited increased CD8^+^ T cell activation, along with elevated viral loads, compared with HLA‐DR15‐negative engrafted animals [71]. Additionally, CD4^+^ T cells derived from these EBV‐infected humanized mice demonstrated cross‐reactivity with MBP‐derived peptides when presented via HLA‐DR15 [71]. These polyreactive T cell responses may be insufficient to adequately control EBV‐infected B cells, which, if left unchecked, could migrate to the CNS and contribute to the development of CNS‐directed autoimmunity.

However, the presence of EBV‐infected B cells in the brains of MS patients remains controversial, with some groups reporting them and others failing to detect them [43, 72, 73]. Nevertheless, EBV‐infected B cells have been shown to adopt a brain‐homing phenotype in mice [74]. Furthermore, a distinct activated T‐bet^+^ CXCR3^+^ memory B cell population could be detected in the brains of MS patients, and CXCR3^+^ B cell neuroinvasiveness was found to correlate with blood EBV viral loads [75]. Recently, our lab provided evidence that primary EBV infection in a humanized mouse model transgenic for the HLA‐DR15 allele and reconstituted with HLA‐DR15‐positive HSCs drives the oligoclonal expansion of a T‐bet^+^ CXCR3^+^ B cell population with neuroinvasive properties. This distinct B cell subset infiltrated the brain in a CXCR3‐dependent manner, thereby facilitating the dissemination of EBV to the CNS. The presence of EBV‐infected B cells within the CNS orchestrated the recruitment of activated effector cells, including CD8^+^ T cells as well as Th1 and Th17 CD4^+^ T cells. This lymphocyte migration to the CNS could be a direct consequence of insufficiently controlled EBV‐infected B cells, particularly given that increased viremia correlated with enhanced CNS infiltration, despite the presence of large numbers of activated CD8^+^ T cells [62]. This might preferentially occur during IM, which is characterized by high numbers of circulating EBV‐infected memory B cells [76]. Within the CNS, these EBV‐infected B cells may stimulate autoreactive CD4^+^ T cells and thereby promote MS pathogenesis, as has been previously observed in MS patients [77]. Understanding the mechanism and role of this potential pathogenic EBV‐infected B cell subset opens new avenues for potential future MS treatments.

### 4.2 Strategies to Target EBV in MS Patients

The strong association between EBV and MS, along with the extremely low risk of MS development in EBV‐seronegative individuals [40], has made EBV a prime target in therapeutic approaches to treat or prevent MS. A suitable prophylactic EBV vaccine could prevent infection or reduce IM incidence, thereby lowering the risk of developing MS. Especially EBV‐seronegative adolescents, who are at an increased risk of developing IM, could particularly benefit from such a vaccine [2]. Most vaccination studies to date focus on the EBV glycoproteins (gp350, gH/gL, gB, and gp42), crucial fusion proteins for viral attachment of B and epithelial cells [78].

While clinical trials demonstrated a lowered incidence rate of IM after immunization with recombinant gp350, the vaccine failed to provide sterilizing immunity [79]. More recently, vaccines targeting additional EBV surface glycoproteins such as the gH/gL‐gp42 heterotrimer and the gH/gL complex, both of which are necessary for viral cell entry, have demonstrated neutralizing and protective effects in preclinical mouse models [80]. Additionally, therapeutic vaccines, initially developed for the treatment of EBV‐associated malignancies, are being explored for the treatment of MS. They utilize a dendritic cell‐based vaccine approach by expressing the EBV latency proteins LMP2 and EBNA1, which aims to boost virus‐specific CD4^+^ and CD8^+^ T cell responses [81]. However, vaccine design must be carefully considered to avoid triggering cross‐reactive immune responses. Immunization with certain EBV antigens, such as EBNA1, might unintentionally increase the risk for MS development [82].

Beyond vaccines, strategies aimed at eliminating EBV‐infected cells have also gained attention. Monoclonal antibodies targeting CD20 are already frequently used in MS therapy and have demonstrated remarkable success in slowing the progression of primary progressive disease [83, 84]. While long unclear how B cell depletion therapy benefited MS treatment, it is now hypothesized that the efficacy may come from the systemic elimination of EBV‐harboring B cells [85]. Indeed, the frequency of EBV‐reactive T cells was decreased following B cell depletion therapy [86]. While it remains unclear to what extent anti‐CD20 depleting therapies can eliminate CNS resident EBV‐infected B cells, our mouse model demonstrated that Rituximab efficiently diminishes CNS viral loads [62]. These CNS resident B cells could have acted as potent APCs, efficiently recruiting and activating CD4^+^ T cells [87, 88]. However, anti‐CD20 therapy is still a form of broad immune depletion and is associated with potential adverse effects [89]. More targeted approaches, such as the specific depletion of the T‐bet^+^ CXCR3^+^ B cell subset or inhibiting the trafficking of EBV‐infected B cells into the CNS, could be more precise therapeutic strategies with reduced treatment‐associated risks [90].

To address the need for greater specificity, various strategies are being explored to uniquely target EBV‐infected B cells. One potential method to eliminate EBV‐infected B cells is the adoptive transfer of autologous EBV‐specific T cells. In a small clinical trial involving MS patients, T cells expanded with an adenoviral vector encoding EBNA1, LMP1, and LMP2A could effectively improve clinical symptoms with reduced disease activity [91]. Another strategy to eliminate EBV‐infected B cells involves the use of agents like histone deacetylase inhibitors, which induce viral reactivation and lytic protein expression. Subsequent treatment with the antiviral drug ganciclovir selectively kills these reactivated cells; in some patients with EBV‐associated malignancies, this approach has led to partial remission [92]. Overall, these diverse strategies underscore EBV as a compelling therapeutic target, with the potential to significantly advance both the prevention and treatment of MS.

## 5 Conclusion

EBV has long been suspected to be involved in MS pathogenesis [35]. This association has been further supported by the fact that virtually all MS patients are EBV‐seropositive and that EBNA1, and, to a lesser extent, LMP1 and EBNA2, can be detected in brain lesions of MS patients [93]. More recently, a 2022 longitudinal study demonstrated that EBV infection in adulthood results in a 32‐fold increased risk to develop MS [40]. Furthermore, recent advances in EBV‐targeting therapeutic strategies further underscore EBV as playing a contributing role in disease progression [83, 91].

Interestingly, the EBV—autoimmunity axis is not restricted to MS, as the virus has been linked to a wide range of autoimmune conditions [94, 95, 96]. In the context of autoimmunity, EBV's lifelong persistence in host B cells, combined with its capacity for periodic reactivation and establishment of chronic inflammation, may create an immune environment that is permissive to the loss of self‐tolerance, particularly in the presence of disease‐specific genetic risk factors, such as HLA‐DR15 in MS [71]. Indeed, many EBV‐associated autoimmune diseases share common features of dysregulated immune control, including dysregulated humoral and cellular responses to the virus, expansion of autoreactive B cells, increased lytic replication, elevated viral loads, and altered EBV‐specific T cell reactivity [42, 94, 96].

Although the exact mechanisms by which EBV contributes to such a wide range of autoimmune diseases remain incompletely understood, several latent and lytic EBV antigens have emerged as candidates with distinct immunomodulatory properties that may contribute to MS pathogenesis (Table 1). For instance, EBV lytic reactivation, resulting in increased expression of immediate early genes, such as BZLF1 and BRLF1, is associated with immune activation and inflammation. Notably, EBV reactivation has been found to coincide with MS relapses [108]. Second, viral antigenic molecular mimicry against EBNA1, BHRF1, or BPLF1 could trigger or sustain autoreactivity. Of particular interest is EBNA1, which shares homology with multiple CNS antigens. Indeed, EBNA1‐specific CD4^+^ T cells and EBNA1 targeting antibodies have been demonstrated to be cross‐reactive with CNS antigens, particularly in individuals carrying the HLA‐DR15 haplotype [44, 103]. Lastly, EBV's latent EBNA2 protein has been shown to bind to MS risk loci, promoting B cell transformation and proliferation and exploiting genetic MS susceptibility [109].

**TABLE 1:** Overview of Immunopathogenic mechanisms of selected lytic and latent EBV genes.

EBV genes		Immunopathogenic mechanisms linking EBV genes to MS
Lytic	BZLF1	Lytic reactivation, immune activation, and inflammatory flares [97, 98]
	BRLF1	Increases expression of pro‐inflammatory cytokines IL‐6 and IL‐1β, leading to potential progression of MS [99]
	BHRF1	Molecular mimicry associated with HLA‐DR‐derived self‐peptides [77, 100]
	BPLF1	Molecular mimicry associated with HLA‐DR‐derived self‐peptides [77, 101]
Latent	EBNA1	Elevated in MS brain lesions, EBNA1^+^ cells found in closer proximity to neurons [93], molecular mimicry associated with MBP [44], anoctamin 2 [102], glial cell adhesion molecule [103], and α‐crystallin B [104]
	EBNA2	Binding to MS risk loci, alteration of host immune response in the presence of MS risk alleles [105]
	LMP2A	Overrepresented in brains of MS patients, modifies activation and survival state of host B lymphocytes [106]
	LMP1	LMP1‐expressing B cells found in brain of MS patients promote survival and immortalization of B cells [107]

However, these mechanisms are unable to fully explain how EBV might initiate or sustain MS. The recently described EBV‐driven, oligoclonal expansion of an inflammatory B cell subset offers a direct link between EBV transformation of B cells and MS initiation. In HLA‐DR15 transgenic, humanized mice, primary EBV infection was shown to expand a pro‐inflammatory T‐bet^+^ CXCR3^+^ B population with CNS homing capacity and the ability to recruit inflammatory T cells. Furthermore, these cells show downregulation of EBV genes, suggesting effective immune evasion by entering latency 0, and may represent a subset of latently infected B cells poised for lytic reactivation [62]. Once reactivated, their plasmablast progeny could generate oligoclonal antibodies with the potential to cross‐react with EBNA1 and various CNS antigens. Lytic reactivation in these cells may also enhance antigen uptake and presentation to inflammatory T cells, strengthening local immune activation within the CNS.

Overall, current evidence supports a model in which EBV contributes to MS through the combined effects of various latent and lytic genes, which interact with pre‐existing risk factors and may drive or reinforce immune dysregulation, loss of tolerance, as well as the differentiation of B cells toward a pro‐inflammatory, CNS‐homing subset. These T‐bet^+^ CXCR3^+^EBV‐infected B cells may be the missing link that can describe the interplay between EBV latency, lytic reactivation, and how these factors converge with host genetic risk alleles to create “the perfect storm” that facilitates MS initiation and drives disease progression.

## Conflicts of Interest

The authors declare no conflicts of interest.

## Data Availability

Data sharing is not applicable to this article as no datasets were generated or analyzed during the current study.
